# CFTR Inhibitors Display Antiviral Activity against Herpes Simplex Virus

**DOI:** 10.3390/v16081308

**Published:** 2024-08-16

**Authors:** Ping Jiang, Zhong Dai, Chan Yang, Liqiong Ding, Songshan Li, Xinfeng Xu, Chen Cheng, Jinshen Wang, Shuwen Liu

**Affiliations:** 1 Guangdong Provincial Key Laboratory of New Drug Screening, Guangzhou Key Laboratory of Drug Research for Emerging Virus Prevention and Treatment, School of Pharmaceutical Sciences, Southern Medical University, Guangzhou 510515, China; 2 Guangdong Key Laboratory for Research and Development of Natural Drugs, Dongguan Key Laboratory of Traditional Chinese Medicine and New Pharmaceutical Development, School of Pharmacy, Guangdong Medical University, Dongguan 523808, China; 3 School of Pharmaceutical Sciences, Hubei University of Science and Technology, Xianning 437100, China; 4 State Key Laboratory of Organ Failure Research, Guangdong Provincial Institute of Nephrology, Southern Medical University, Guangzhou 510515, China

**Keywords:** herpes simplex virus (HSV), cystic fibrosis transmembrane conductance regulator (CFTR), Glyh-101, IOWH-032, CFTRi-172

## Abstract

The cystic fibrosis transmembrane conductance regulator (CFTR), a cAMP-dependent Cl^−^ channel, is closely associated with multiple pathogen infections, such as SARS-CoV-2. However, whether the function of the CFTR is involved in herpes simplex virus (HSV) infection has not been reported. To evaluate the association of CFTR activity with HSV infection, the antiviral effect of CFTR inhibitors in epithelial cells and HSV-infected mice was tested in this study. The data showed that treatment with CFTR inhibitors in different concentrations, Glyh-101 (5–20 μM), CFTRi-172 (5–20 μM) and IOWH-032 (5–20 μM), or the gene silence of the CFTR could suppress herpes simplex virus 1 (HSV-1) and herpes simplex virus 2 (HSV-2) replication in human HaCaT keratinocytes cells, and that a CFTR inhibitor, Glyh-101 (10–20 μM), protected mice from HSV-1 and HSV-2 infection. Intracellular Cl^−^ concentration ([Cl^−^]_i_) was decreased after HSV infection via the activation of adenylyl cyclase (AC)-cAMP signaling pathways. CFTR inhibitors (20 μM) increased the reduced [Cl^−^]_i_ caused by HSV infection in host epithelial cells. Additionally, CFTR inhibitors reduced the activity and phosphorylation of SGK1 in infected cells and tissues (from the eye and vagina). Our study found that CFTR inhibitors can effectively suppress HSV-1 and HSV-2 infection, revealing a previously unknown role of CFTR inhibitors in HSV infection and suggesting new perspectives on the mechanisms governing HSV infection in host epithelial cells, as well as leading to potential novel treatments.

## 1. Introduction

Herpes simplex virus (HSV) is a member of the Herpesviridae family and the Alphaherpesvirinae subfamily, including Herpes simplex virus 1 (HSV-1) and Herpes simplex virus 2 (HSV-2) [1], which are highly prevalent human pathogens with worldwide prevalence levels of about 67% and 13%, respectively [2]. Herpes simplex virus had been demonstrated to manipulate ion channels to facilitate viral infection. For example, HSV-1 glycoprotein D interacted with the nonselective cation channel, the transient receptor potential canonical 1 (TRPC1) [3] and the transient receptor potential vanilloid 2 (TRPV2) [4]. These interactions facilitated viral entry in a manner dependent on Ca^2+^ signaling pathways. Knockout of TRPC1 or TRPV2 prevented viral infection and protected mice from HSV-1 infection [3,4]. HSV-1 infection significantly reduced the expression of the voltage-gated sodium channels (VGSCs) and completely abolished VGSC activity on the dorsal root ganglia (DRG) of neurons [5]. HSV-2 infection was associated with the T-type calcium channel. The blocking of the T-type calcium channel inhibited HSV-2 infection at the late stage of genome replication [6]. In addition to these cation channels, HSV infection is also strongly associated with the volume-regulated anion channel (VRAC), which controls cell volume by releasing chloride and organic osmolytes in response to cell swelling [7]. Two well-known VRAC inhibitors showed antiviral defense against HSV-1 infection [8]. These reports suggested that multiple ion channels participated in the HSV infection.

The cystic fibrosis transmembrane conductance regulator (CFTR) is a crucial cAMP-dependent anion channel for the main transport of chloride ions [9]. It had been reported that the CFTR dynamically modulated the intracellular Cl^−^ concentration ([Cl^−^]_i_) to regulate host immune defenses [10,11] and viral infection [12,13]. An investigation indicated that the CF patients who suffered from a CFTR monogenic mutation improved their survival after contracting COVID-19 [14]. Dysfunctional CFTR altered the susceptibility to severe acute respiratory syndrome coronavirus-2 (SARS-CoV-2) infection, resulting in the reduced viral entry and replication in cystic fibrosis cells [15], and CFTR inhibitors displayed antiviral activity against SARS-CoV-2 in vitro [16]. Severe influenza virus (IAV) infections destroyed the CFTR activity in the respiratory and alveolar epithelial cells [12]. BK polyomavirus (BKPyV) invasion necessitated that the CFTR activity deliver virions into the endoplasmic reticulum (ER) [13]. The function of the CFTR was inhibited in HIV-infected epithelial cells [17]. The above investigations suggested that CFTR activity participated in various viral infections. However, it is not clear whether its activity is involved in HSV-1 and HSV-2 infection.

In our previous study, we had screened a compound library with 362 ion channel modulators (TargetMol, MA, USA) and had found that a specific CFTR inhibitor, IOWH-032, had antiviral effects against HSV-2 [6], raising our interest in the relationship between CFTR activity and HSV infection.

In this study, we demonstrated that HSV-1 and HSV-2 infections induced the adenylyl cyclase (AC)–cAMP pathway to activate the cAMP-dependent Cl^−^ channel (CFTR) in epithelial cells. Knockdown of CFTR in cells or the inhibition of CFTR activity by pharmacological inhibitors suppressed viral infection and protected mice from HSV-1 and HSV-2 infection. Our results revealed a previously unknown function of the CFTR in HSV-1 and HSV-2 infection.

## 2. Materials and Methods

### 2.1. Cells, Viruses, and Mice

Human HaCaT keratinocytes cells and Vero cells were purchased from ATCC (Manassas, VA, USA) and cultured in DMEM with high glucose (Gobio, Malvern, PA, USA), 10% fetal bovine serum (Gibco, Life Technologies), and 1% gentamicin (Sigma, Setagaya, Japan). The HSV-2 333 strain was provided by the Guangzhou Institute of Biomedicine and Health (Guangzhou, China). The HSV-1 F strain was provided by the College of Pharmacy at Jinan University (Guangzhou, China).

Female BALB/c mice at the age of seven weeks were purchased from Vital River Laboratories in Guangzhou, China. The animals were given ad libitum access to food and water in a room maintained at a constant temperature of 24 ± 0.5 °C with a 12:12 h light/dark photoperiod.

### 2.2. Reagents and Antibodies

Glyh-101 (219671), CFTR_inh_-172 (CFTRi-172, C2992), and methylcellulose were purchased from Sigma-Aldrich. IOWH-032 (HY-18337) and GSK 650394 (HY-15192) were purchased from MedChemExpress (MCE, Monmouth Junction, NJ, USA). Acyclovir (S24717) was purchased from Shanghai Yuanye Biotechnology Co., Ltd. (Shanghai, China). The Human AC ELISA Kit (SU-BN18654) and the Human cGAS ELISA Kit (SU-BN16201) were purchased from Rising Biotechnology Co., LTD (Quanzhou, Fujian, China). The Cyclic AMP ELISA Kit (Item No. 581001) was purchased from Cayman Chemical (Ann Arbor, MI, USA). A SGK1 Kinase Enzyme System was purchased from Promega (V9671, Madison, WI, USA). Lipofectamine™ 3000 was purchased from Invitrogen (Waltham, MA, USA). The anti-GAPDH antibody was purchased from Cell Signaling Technology (Danvers, MA, USA). The anti-HSV1+HSV2 gD antibody (ab6507) was purchased from Abcam (Cambridge, UK). MQAE (E3101) was purchased from ThermoFisher (Waltham, MA, USA).

### 2.3. Plaque Assay

A plaque assay was used to measure viral titer, which is related to the virus infectivity [6]. Vero cells were inoculated in 6-well plates with a density of 2.0 × 10^5^ cells per well. Diluted supernatants of infected cells were added to the Vero cells. After incubation for 1 h at 37 °C, the culture supernatants were discarded, and the DMEM medium containing 2% fetal bovine serum and 1% methylcellulose was added. After incubation for 3–4 days at 37 °C, crystal violet was used to stain the cells. The plaques were filmed with an enzyme-linked immunoblot analyzer to calculate the yield of the progeny virus in the supernatant.

### 2.4. Real-Time Quantitative PCR

Total RNA was extracted using a total RNA isolation kit. RNA concentrations were measured by NanoDrop 2000c (Thermo Scientific, Waltham, MA, USA). cDNA was synthesized through reverse transcription and gently mixed with ChamQ SYBR qPCR Master Mix and primers. Mixtures were performed on the LightCycler 480 system (Roche, Basel, Switzerland). The primer sequences are listed in Table 1. The levels of target genes were normalized to the levels of GAPDH mRNA, and relative expression was determined according to the ΔΔCt method [18].

### 2.5. Western Blot Assay

The total protein was extracted with the pre-cooled RIPA buffer (Bio-Rad, Hercules, CA, USA). The protein concentration was detected using the BCA protein assay kit (KWBIO, kw0014, Beijing, China). After being separated by SDS-polyacrylamide gel electrophoresis (SDS-PAGE), the protein samples were transferred to polyvinylidene fluoride membranes (Millipore, Burlington, MA, USA). The membranes were blocked with 5% fat-free milk for 1 h at room temperature, then incubated with the primary antibody at 4 °C overnight, and finally incubated with the secondary antibody at room temperature for 1 h. The SuperSignal ECL reagent (Millipore, Burlington, MA, USA) was used to observe the chemiluminescence signals using a multifunctional imaging system (ProteinSimple, San Jose, CA, USA) [19].

### 2.6. Immunofluorescence

Immunofluorescence staining was performed as previously described [6]. The infected cells, vaginal tissues, and eye tissues were fixed with acetone at 4 °C for 10 min.

After washing with phosphate-buffered saline (PBS) and treating with 1% BSA for 30 min at room temperature, the samples were incubated with the first primary antibody at 4 °C overnight, followed by the secondary antibody, goat anti-mouse IgG conjugated to fluorescein isothiocyanate (FITC) or Texas red (Santa Cruz Biotechnology, Dallas, TX, USA), at room temperature for one hour. VECTA-SHIELD medium (Vector Labs., Burlingame, CA, USA) was used to preserve the samples. Finally, images were captured via a fluorescence microscope (TE2000U, Tecan, Morrisville, NC, USA).

### 2.7. Virus Infection

The cells were inoculated in 6-well plates at a density of 1 × 10^6^ cells per well. The next day, HSV-1 (MOI = 1) or HSV-2 (MOI = 1) was administered to the cells in the presence or absence of an inhibitor for 1 h. Subsequently, the unabsorbed virus was removed, and DMEM containing an inhibitor was added [6]. The cells were collected at the specified time points for quantitative PCR, Western blot, and immunofluorescence staining. The culture supernatants were collected for plaque assays.

### 2.8. Mouse Model of Vaginal HSV-2 Infection

All animal experiments were conducted following protocols approved by the Animal Use and Care Committee of Southern Medical University. Thirty-five female BALB/c mice were divided into five groups according to body weight, including a control group, an HSV-2 infected group, a Glyh-101 low-dose group (15 mg/kg/d), a Glyh-101 high-dose group (30 mg/kg/d), and an acyclovir group (30 mg/kg/d), with *n* = 7 per group.

The vaginal cavities of the mice were washed with PBS and rubbed with sterile cotton to increase susceptibility to HSV-2. The mice were then inoculated vaginally with 10^6^ plaque-forming units (PFU) of the HSV-2 333 strain. The phenotypes of the infected mice were scored daily as follows [20]: “1”, slight genital erythema and edema; “2”, moderate genital inflammation; “3”, severe exudative genital lesions; “4”, hind limb paralysis; or “5”, death. Vaginal tissues were collected for hematoxylin and eosin (HE) staining, immunofluorescence staining, real-time quantitative PCR, and Western blot.

### 2.9. Mouse Model of Ocular HSV-1 Infection

All animal experiments were conducted following protocols approved by the Animal Use and Care Committee of Southern Medical University. Thirty-five female BALB/c mice were divided into five groups according to body weight, including a control group, an HSV-1 infected group, a Glyh-101 low-dose group (15 mg/kg/d), a Glyh-101 high-dose group (30 mg/kg/d), and an acyclovir group (30 mg/kg/d), with *n* = 7 per group.

The cornea was scarified, and each cornea was infected with 10^6^ PFU of the HSV-1 F strain. The phenotypes of the infected mice were scored as follows [3]: “0”, completely transparent cornea; “1”, minimal corneal opacity, but with the iris clearly visible; “2”, moderate corneal opacity, with iris vessels still visible; “3”, moderate corneal opacity, the presence of a pupil margin but with iris vessels not visible; “4”, complete corneal opacity, with the pupil not visible. The mice were sacrificed, and the eyes were collected after seven days of infection for hematoxylin and eosin (HE) staining, immunofluorescence staining, real-time quantitative PCR, and Western blot.

### 2.10. Antiviral Activity Assay

Vero cells were inoculated into 96-well plates with a density of 1 × 10^4^ cells per well. The next day, the cells were treated with 100 TCID_50_ of HSV-1 or HSV-2 and serially diluted compounds in three replicates. After incubation for 72 h, MTT was added to the cells with a concentration of 0.5 mg/mL, followed by incubation for 4 h. The supernatants were discarded and DMSO was added. The plates were placed on a shaker for 15 min until the formazan was completely dissolved. Finally, the absorbance was measured at 570 nm using a microplate reader (Tecan, Switzerland). Antiviral activity was determined using the following formula: Inhibition rate (%) = [OD _(D)_–OD _(V)_]/[OD _(C)_–OD _(V)_] × 100%, where D represents infected cells treated with the compound, V indicates infected cells, and C indicates uninfected cells [19].

### 2.11. Measurement of Intracellular Chloride Concentration ([Cl^−^]_i_) in Cells

The human HaCaT keratinocytes cells were inoculated in 12-well plates at a density of 1.5 × 10^5^ cells per well. After infection with HSV-2 (MOI = 1) or HSV-1 (MOI = 1), cells were washed three times with Hanks’ balanced salt solution (HBSS) at the specified time points. Cells were subsequently loaded with 5 mM of N-(ethoxycarbonylmethyl)-6-methoxyquinolinium bromide (MQAE, ThermoFisher, E3101, Waltham, MA, USA) for 30 min at 37 °C. After washing with HBSS for three times, fluorescence excited at 350 nm was recorded using an imaging system (Olympus, IX83, Tokyo, Japan) [11]. Data are expressed as fluorescence units.

### 2.12. ELISA Assay

Quantities of cyclic adenosine monophosphate (cAMP), adenylyl cyclases (ACs), and SGK1 activity were quantified using a direct enzyme immunoassay kit following the manufacturer’s instructions [11,21].

### 2.13. Statistical Analysis

Experimental data are expressed as the means ± SEMs. Students’ two-tailed *t*-tests were used for two sets of data analysis. Three or more groups were analyzed with one-way ANOVAs followed by Dunnett’s post hoc test using GraphPad Prism 8.0 software (* *p* < 0.05, ** *p* < 0.01, *** *p* < 0.001). Two-tailed *p* values < 0.05 were considered statistically significant.

## 3. Results

### 3.1. CFTR Inhibitors Produce an Antiviral Effect In Vitro

**Figure 1:**
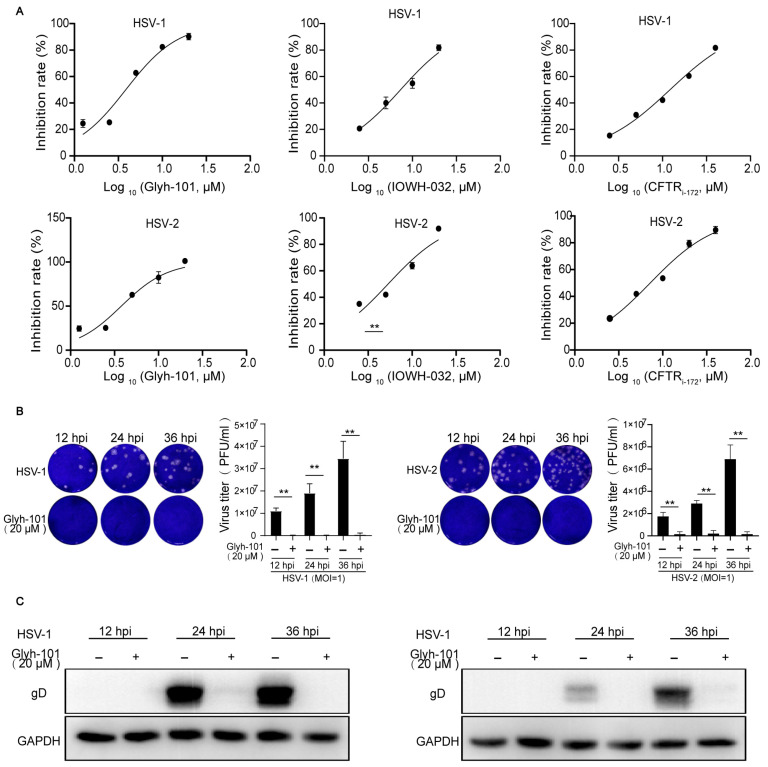
CFTR inhibitors suppress HSV-1 and HSV-2 infection at different post-infection times in vitro. (**A**) The dose-dependent suppression effects of CFTR inhibitors against HSV-1 and HSV-2 infection. Vero cells were, respectively, infected with HSV-1 (MOI = 1) or HSV-2 (MOI = 1) in the presence of the indicated concentrations of CFTR inhibitors for 72 h before conducting MTT cell proliferation assays. (**B**) The suppression effects of CFTR inhibitors on the production of the progeny virus. HaCat cells were, respectively, infected with HSV-1 (MOI = 1) or HSV-2 (MOI = 1) in the presence or absence of Glyh-101 (20 μM) for the indicated amounts of time. The supernatants were harvested from HSV-1-infected cells and were diluted 1 × 10^7^-fold for virus titer by plaque assay at 12, 24, and 36 h post treatment (hpi). The supernatants were harvested from HSV-2-infected cells and diluted 1 × 10^6^-fold for plaque assay at the indicated hours. Representative images of plaque assays are shown on the left. Quantifications of viral titers are shown on the right. (**C**) The suppression effects of CFTR inhibitors on viral protein expression. HaCat cells were, respectively, infected with HSV-1 (MOI = 1) or HSV-2 (MOI = 1) in the presence or absence of Glyh-101 (20 μM) for the indicated hours. The cells were collected for the expression of viral glycoprotein D (gD) at 12, 24, and 36 h post treatment. The data represent three independent experiments and the mean ± SD. The *p*-values are defined as ** *p* < 0.01 vs. infected cells.

We had screened a compound library with 362 ion channel modulators (TargetMol Chemicals Inc., Boston, MA, USA) by measuring their inhibitory effect on the HSV-2-induced cytopathic effect in Vero cells in our previous study. We found that IOWH-032, a specific CFTR inhibitor [22], had apparent antiviral effects at 20 μM [6]. This result raised our hypothesis that CFTR function might be associate with HSV-2 infection. To investigate whether CFTR activity is required for HSV infection, we first evaluated the dose-dependent inhibitory effects of the well-known CFTR inhibitors, CFTRi-172 [23], IOWH-032 [22], and Glyh-101 [24], against HSV-1 and HSV-2 by measuring their cytopathic effect in Vero cells. The results showed that three inhibitors could suppress HSV-1 and HSV-2 infection in a dose-dependent manner (Figure 1A). As Glyh-101 showed the most potent inhibitory effect against HSV-1 and HSV-2 infection in Figure 1A, we picked it out as a representative of a specific CFTR inhibitor in the follow-up experiments. To verify the antiviral effect of CFTR inhibitors on HSV-1 and HSV-2 infection, the synthesis of a viral glycoprotein D (gD) and a viral titer in a cell supernatant were, respectively, analyzed via Western blot and plaque assay at different post-infection times. Human HaCaT cells were, respectively, infected with HSV-1 (MOI = 1) or HSV-2 (MOI = 1) in the presence or absence of Glyh-101 (20μM). The supernatants were harvested and diluted for viral titer testing via plaque assay, and the cells were collected for viral glycoprotein D (gD) testing via Western blot at indicated times. We observed that Glyh-101 visibly inhibits the production of progeny virus (Figure 1B) and the synthesis of viral glycoprotein D (Figure 1C) at 12, 24, and 36 h post-infection (hpi). These results indicated that Glyh-101 could suppress HSV-1 and HSV-2 infection at different post-infection times in human epithelial cells.

### 3.2. Validation of the Antiviral Effects of CFTR Inhibitors In Vitro

**Figure 2:**
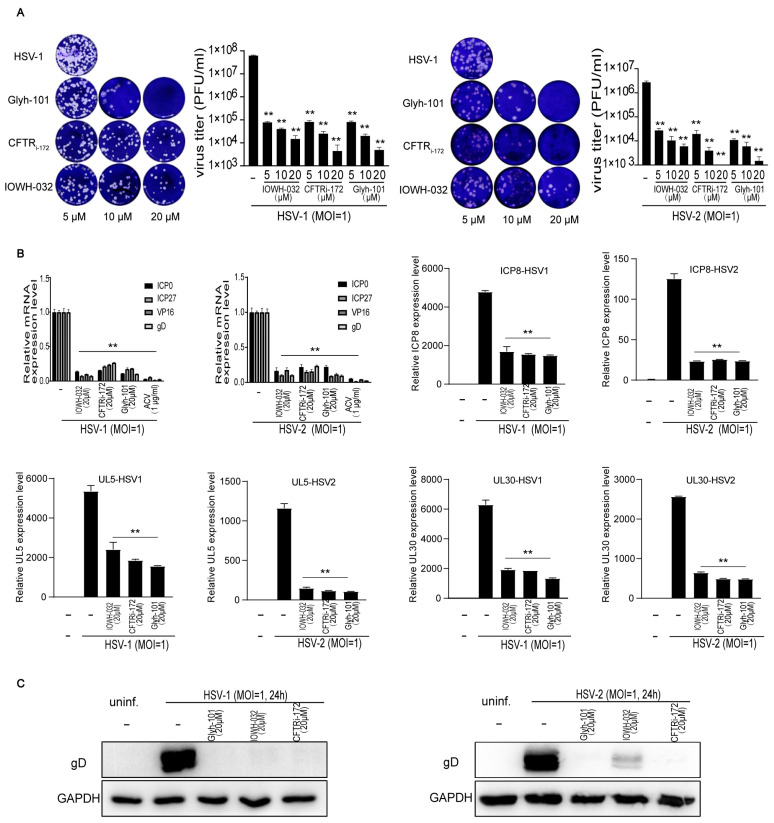
CFTR inhibitors in different concentrations suppress HSV-1 and HSV-2 infection in vitro. HaCat cells were, respectively, infected with HSV-1 (MOI = 1) or HSV-2 (MOI = 1) in the presence or absence of CFTR inhibitors in indicated concentrations for 24 h. (**A**) The supernatants were harvested from HSV-1-infected cells and were diluted 1 × 10^7^-fold for plaque assay to detect viral titers. The supernatants were collected from HSV-2-infected cells and were diluted 1 × 10^6^-fold for plaque assay. Representative images of plaque assay are shown one the left. Quantifications of viral titers are shown one the right. (**B**) HaCat cells were, respectively, infected with HSV-1 (MOI = 1) or HSV-2 (MOI = 1) in the presence or absence of CFTRi-172 (20 μM), IOWH-032 (20 μM), Glyh-101 (20 μM), or acyclovir (ACV; 1 μg/mL) separately for 24 h. The cells were collected for the expressions of the viral genes (ICP0, ICP27, gD, VP16, ICP8, UL5, and UL30) measured by real-time quantitative PCR, and (**C**) the cells were collected for the synthesis of viral glycoprotein D testing via Western blot. Acyclovir, the typical drug used to treat HSV infection, served as the positive control [25]. Data represent the mean ± SD of three independent experiments. The *p*-values are defined as ** *p* < 0.01 vs. infected cells.

To confirm the antiviral effect of CFTR inhibitors in different concentrations in Human HaCaT cells, we performed a plaque assay to measure the production of the progeny virus, Western blot assay to analyze the synthesis of viral glycoprotein D (gD), and Real-time quantitative PCR to detect the expression of viral genes. We discovered that CFTRi-172 (5, 10, 20 μM), IOWH-032 (5, 10, 20 μM), and Glyh-101 (5, 10, 20 μM) visibly reduced the production of the progeny virus (Figure 2A), the expression of viral genes (ICP0, ICP27, VP16, gD, ICP8, UL30, and UL5) (Figure 2B) and the synthesis of gD (Figure 2C), suggesting that CFTR inhibitors could suppress HSV-1 and HSV-2 infection in different concentrations in vitro.

**Figure 3:**
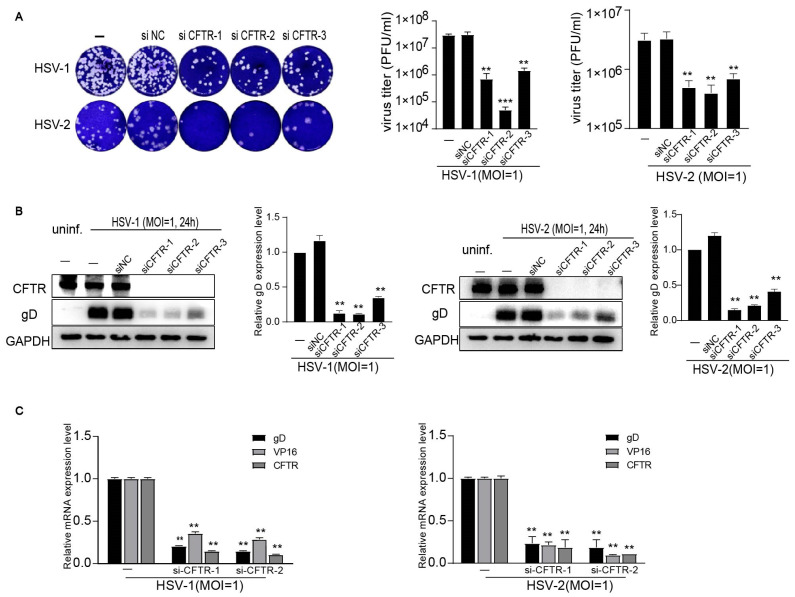
Knockdown of CFTR inhibits HSV-1 and HSV-2 infection in vitro. After transfection with siRNA, HaCat cells were, respectively, infected with HSV-1 (MOI = 1) or HSV-2 (MOI = 1) for 24 h. (**A**) The supernatants were harvested and diluted for the virus titer via plaque assay. (**B**) The cells were collected to analyze viral glycoprotein D expression via Western blot. (**C**) The expression of the viral genes (gD and VP16) and CFTR was measured by real-time quantitative PCR. The data represent three independent experiments and the mean ± SD. The *p*-values are defined as ** *p* < 0.01 and *** *p* < 0.001vs. infected cells.

To further determine the participation of CFTR in HSV infection, we observed the antiviral effect of small interfering RNA (siRNA) targeting against the CFTR in HSV-1 and HSV-2 infections in Human HaCaT cells. The protein and mRNA expression of CFTR was reduced effectively in transfected cells with the siRNA (Figure 3B,C). The siRNA-transfected group had fewer progeny viruses (Figure 3A), viral proteins gD (Figure 3B), and viral genes (Figure 3C) than the un-transfected group, showing that the knockdown of CFTR by gene silence also suppresses the HSV-1 and HSV-2 infection in host epithelial cells.

### 3.3. CFTR Inhibitor Protects Mice from HSV Infection

**Figure 4:**
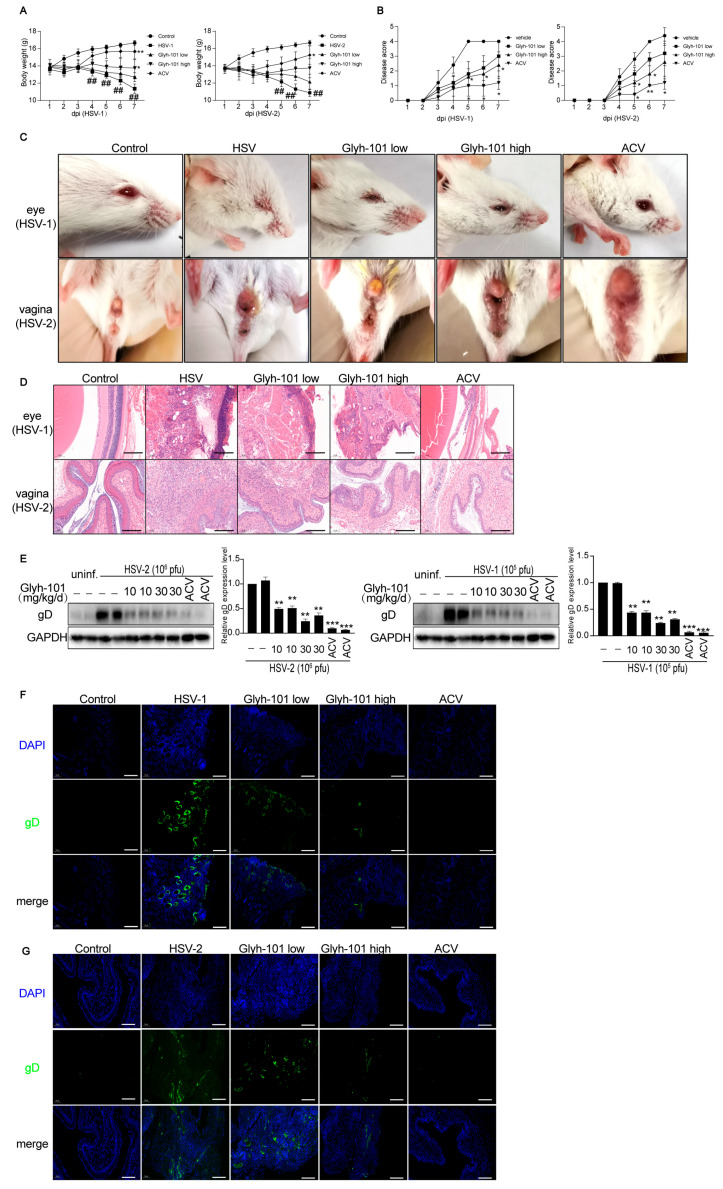
Glyh-101 protects mice against HSV-1 and HSV-2 infection. The mice were infected with 10^5^ plaque-forming units (pfu) per mouse of the HSV-1 F strain or 10^6^ pfu per mouse of the HSV-2 333 strain. The antiviral effects of CFTR inhibitors were evaluated in vivo. (**A**) The weights of the mice were recorded daily. (**B**) Disease scores were observed daily. (**C**) The figures show the representative appearances of the eye or vagina after six days of infection. (**D**) After seven days of infection, the eyes and vaginas were collected and stained with hematoxylin and eosin. The figures show the representative eye and vaginal morphology. (**E**) The eye tissues (**left**) and vaginal tissues (**right**) were collected from infected mice to analyze viral glycoprotein D expression via Western Blot. (**F**) The eye tissues were collected for immunofluorescence staining to evaluate viral infection after seven days of infection. The figures show the representative eye morphology caused by HSV-1 infection. (**G**) The vaginal tissues were observed via immunofluorescence staining after seven days of infection. The figures show the representative vagina morphology caused by HSV-2 infection. *n* = 7 for each infected group. Data represent the mean ± SD. The *p*-values are defined as ## *p* < 0.01 vs. control group. The *p*-values are defined as * *p* < 0.05, ** *p* < 0.01, and *** *p* < 0.001 vs. infected group. Scale bar is 200 μm.

To examine the antiviral effect of CFTR inhibitors in infected mice, we established an ocular infection model using the HSV-1 F strain and a vaginal infection model using the HSV-2 333 strain in WT BALB/c mice. We observed that the weights of animals in the vehicle treatment group decreased gradually every day. However, the reduction in weight was greatly ameliorated in the Glyh-101 high-dose (30 mg/kg/d) treatment group and the ACV (30 mg/kg/d) treatment group (Figure 4A). Quantitatively, the infected mice in the Glyh-101 treatment group had lower disease scores than those in the vehicle treatment group (Figure 4B).

The HSV-1 F strain causes edema in the eyeballs with corneal opacity, even causing the pupil to not be visible [26,27]. The HSV-2 333 strain leads to exudative genital lesions and hind limb paralysis [26,28]. Overall observation showed that the pupil of HSV-1-infected mice was not visible, and the cornea appeared to have severe opacity in HSV-1-infected mice. Mild corneal opacity was observed in the Glyh-101 low-dose (15 mg/kg/d) treatment group, edema and corneal opacity were not presented in the Glyh-101 high-dose (30 mg/kg/d) treatment group (Figure 4C, above), suggesting that Glyh-101 treatment protected the eye from HSV-1 infection. Overall observation pointed out that vaginal exudation distinctly increased in HSV-2 infected mice, and Glyh-101 intervention significantly reduced vaginal exudation both in the Glyh-101 low- and high-dose groups (Figure 4C, below), suggesting that Glyh-101 treatment protected the vagina from HSV-2 infection.

The eyes and vagina were collected for Hematoxylin and eosin (HE) staining, Western blot, and immunofluorescence assays to evaluate the protective effect of Glyh-101 in infected mice. HE staining (Figure 4D) showed that HSV-1 and HSV-2 infection, respectively, results in inflammatory cell infiltration in the iris or in the lamina propria, the muscular layer of vagina. Glyh-101 treatment decreased the infiltration of inflammatory cells in HSV-1- and HSV-2-infected mice meaningfully (Figure 4D).

Western blot analysis indicated that Glyh-101 treatment restrains the synthesis of gD in HSV-1- and HSV-2-infected mice (Figure 4E). The fluorescence analysis suggested that the green fluorescence of HSV-1 glycoprotein D (Figure 4F) and HSV-2 glycoprotein D (Figure 4G) mainly emerged in HSV-1- or HSV-2-infected tissues. Glyh-101 treatment visibly lessened the green fluorescence of gD in infected mice, especially in the Glyh-101 high-dose group (Figure 4F,G). Acyclovir (ACV), the typical drug used to treat HSV-1 and HSV-2 infection, served as the positive control [25]. Taken together, these data indicated that Glyh-101, a specific CFTR inhibitor, protected mice against HSV-1 and HSV-2 infection.

### 3.4. HSV Infection Activates CFTR-Mediated Cl^−^ Transport Activity

**Figure 5:**
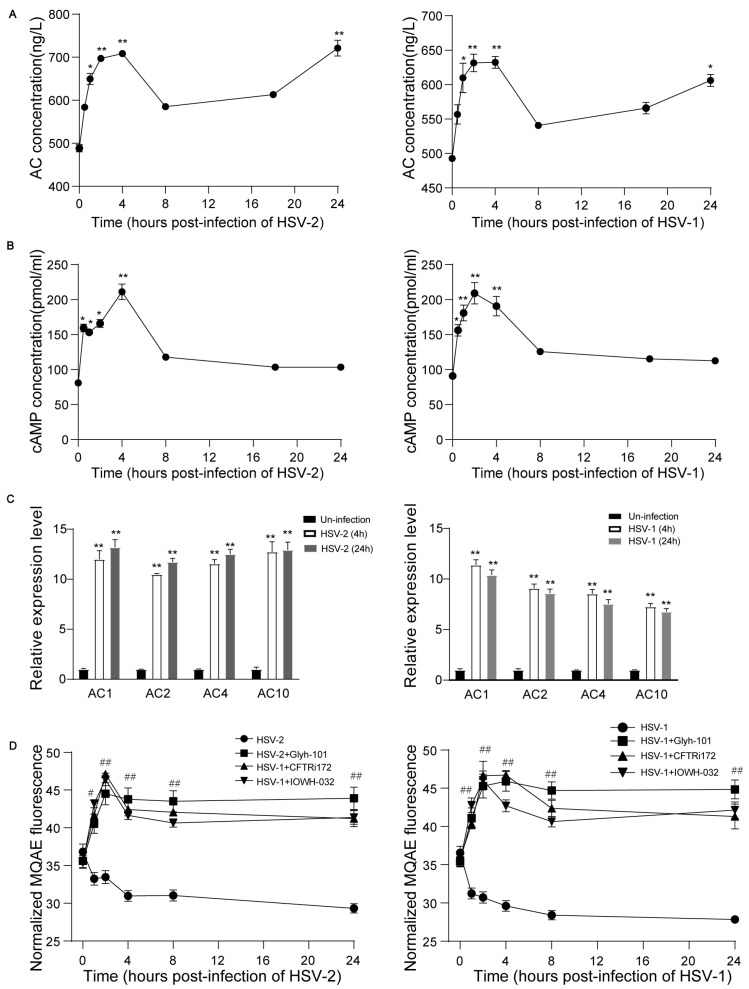
HSV infection activates CFTR-mediated Cl^−^ transport activity via the up-regulation of AC-cAMP signaling. HaCaT keratinocytes cells were infected with HSV-1 (MOI = 1) or HSV-2 (MOI = 1) at different time points, and the whole-cell lysates were harvested to detect the concentration of ACs (**A**) and cAMP (**B**) by ELISA. (**C**) HaCaT cells were infected with HSV-1 (MOI = 1) or HSV-2 (MOI = 1) for 4 or 24 h, the cells were collected for AC1-10 mRNA expression analysis by real-time quantitative PCR. (**D**) Alterations in [Cl^−^]_i_ after HSV-1 (MOI = 1) or HSV-2 (MOI = 1) infection in HaCaT keratinocytes cells. Data represent the mean ± SD. The *p*-values are defined as * *p* < 0.05 and ** *p* < 0.01 vs. uninfected cells. The *p*-values are defined as ^#^ *p* < 0.05 and ^##^ *p* < 0.01 vs. infected cells.

The cystic fibrosis transmembrane conductance regulator (CFTR) is a cyclic adenosine monophosphate (cAMP)-dependent Cl^−^ channel [9]. Intracellular cAMP is generated from ATP by adenylyl cyclases (ACs) [29]. Therefore, we investigated whether AC-cAMP signaling was changed as induced by HSV infection. The concentrations of AC and cAMP were tested via ELISA. We found that the AC concentrations were markedly increased after HSV-1 and HSV-2 infection at each time point (Figure 5A), and the cAMP concentrations were also increased markedly at 0.5, 1, 2, and 4 h after infection with HSV-1and HSV-2 (Figure 5B).

It is well known that two different classes of ACs including hormonal and G-protein-regulated enzymes with a transmembranous component (tmAC) and soluble adenylyl cyclase (sAC) are responsible for generating intracellular cAMP [29,30]. In mammals, the tmAC class contains nine members transcribed from nine different genes [31]. In order to further investigate which ACs are implicated in HSV infection, the mRNA expressions of AC members (AC1–AC10) were detected via QRT-PCR. The data indicated that HSV infection increased the mRNA expressions of AC1, AC2, AC4, and sAC (AC10) at 4 and 24 h (Figure 5C).

The CFTR mainly modulates the intracellular Cl^−^ concentration ([Cl^−^]_i_) in epithelial cells [9,32]. The alteration in [Cl^−^]_i_ after HSV infection in cells was tested by fluorochrome MQAE. Intriguingly, we observed that [Cl^−^]_i_ was decreased gradually in HSV-1- and HSV-2-infected cells, and CFTR inhibitors increased the [Cl^−^]_i_ markedly at different time points (Figure 5D). Collectively, these results indicated that HSV infection up-regulated the expression of AC1, AC2, AC4, sAC, and intracellular cAMP to activate CFTR activity, resulting in a decrease in [Cl^−^]_i_. CFTR inhibitor suppressed the CFTR-mediated Cl^−^ transport activity to elevate [Cl^−^]_i_ in infected cells.

### 3.5. CFTR Inhibitors Reduce SGK1 Activity in Infected Cells and Tissues

**Figure 6:**
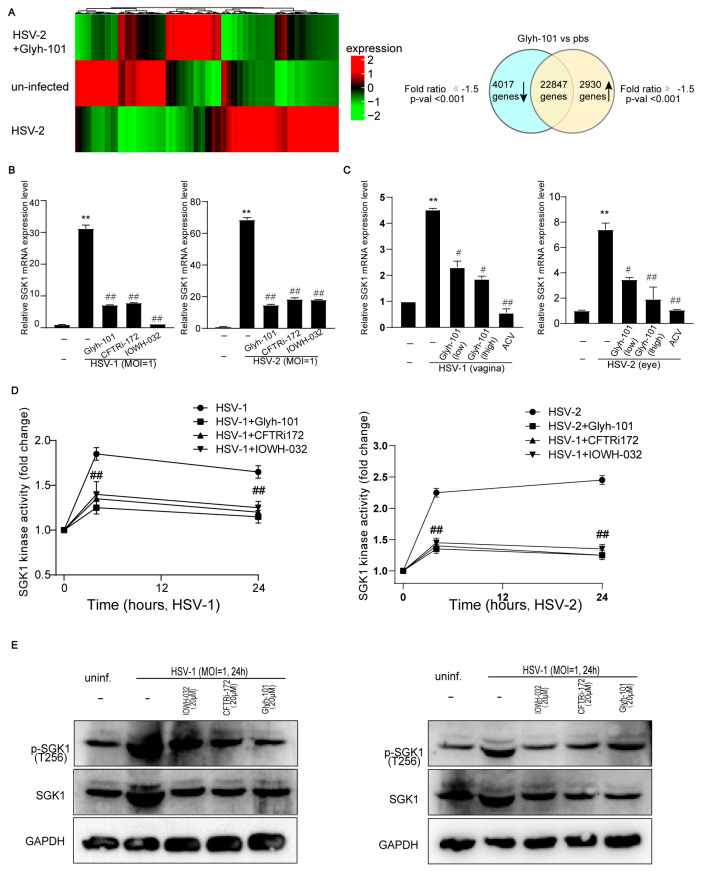
CFTR inhibitors reduce SGK1 activity in infected cells. (**A**) HaCaT keratinocytes cells were infected with HSV-2 (MOI = 1) for 24 h in the presence or absence of Glyh-101 (20 μM), and the whole-cell lysates were harvested for an RNA-sequencing analysis. (**B**) The SGK1 mRNA expression was tested via real-time quantitative PCR in infected cells. (**C**) The SGK1 mRNA expression was tested in infected tissues (from eyes and vaginas). (**D**) HaCaT keratinocytes cells were infected with HSV-2 (MOI = 1) or HSV-1 (MOI = 1) in the presence or absence of Glyh-101 (20 μM), CFTRi-172 (20 μM), and IOWH-032 (20 μM) for 4 or 24 h, and the cells were harvested for SGK1 kinase activity assessing via a SGK1 Kinase Enzyme System. (**E**) Western Blot images showing SGK1 phosphorylation in infected cells. Data represent the mean ± SD. The *p*-values are defined as ** *p* < 0.01 vs. uninfected cells. The *p*-values are defined as ^#^ *p* < 0.05 and ^##^ *p* < 0.01 vs. infected cells.

To further investigate the mechanism of antiviral effects of CFTR inhibitors, we presented the related gene expression profile and signaling pathways by performing an RNA-sequencing analysis. Using an analysis of variance obtained from 29,794 genes, we identified a subset of 6947 differentially expressed genes. Of these genes, 2930 were up-regulated, and 4017 were down-regulated (Figure 6A). Some molecules which are Cl^−^-sensitive genes were selected to analyze the mechanisms of antiviral effects of CFTR inhibitors.

We focused on the fact that the changes in serum and glucocorticoid-inducible protein kinase 1 (SGK1) mRNA expression were noticeable in the RNA-sequencing analysis. Knowing that SGK1 is a Cl^−^-sensitive kinase [11,33,34,35] suggests that SGK1 might be regulated by CFTR inhibitors and could be implicated in HSV infection. Our in vitro study demonstrated that the SGK1 mRNA expression was up-regulated in infected cells, and the CFTR inhibitors, Glyh-101, CFTRi-172, and IOWH-032, suppressed SGK1 mRNA expression in infected cells (Figure 6B). Glyh-101 also suppressed SGK1 mRNA expression in infected eyes and vaginas (Figure 6C).

Since SGK1 is a serine–threonine kinase, phosphorylation at the threonine 256 locus was mandatory for the maximal activation of SGK1 [33]. Our results found that the SGK1 activity was up-regulated at 4 and 24 h post-infection, and the CFTR inhibitors, Glyh-101, CFTRi-172, and IOWH-032, attenuated the SGK1 activity in infected cells (Figure 6D). There was a higher content of SGK1 expression and phosphorylation in infected cells. The CFTR inhibitors, Glyh-101, CFTRi-172, and IOWH-032, suppressed the synthesis and phosphorylation of SGK1 (Figure 6E). Taken together, HSV infection heightened CFTR-mediated Cl^−^ transport activity which subsequently activated SGK1; CFTR inhibitors suppressed the CFTR-mediated Cl^−^ transport activity to reduce SGK1 activity in infected cells.

## 4. Discussion

The cystic fibrosis transmembrane conductance regulator (CFTR) is a cAMP-dependent anion channel mainly used for the transport of chloride [9]. The function of the CFTR in herpes simplex virus infection has not been reported. In this study, we discovered that the CFTR inhibitors, Glyh-101, IOWH-032, and CFTRi-172, or the gene silence of the CFTR could suppress herpes simplex virus 1 (HSV-1) and herpes simplex virus 2 (HSV-2) replication in human HaCaT keratinocytes cells, and that a CFTR inhibitor, Glyh-101, protected mice from HSV-1 and HSV-2 infection. This is consistent with previous studies, reporting that non-selective chloride channel inhibitors, including tamoxifen and 5-nitro-2-(3-phenylpropylamino)-benzoate (NPPB), significantly inhibited HSV-1 infection [36]. The data suggested that the functioning of the CFTR participates in HSV-1 and HSV-2 infection in host epithelial cells.

We observed that Glyh-101 can inhibit the infection of herpes simplex virus visibly in vivo. But, the antiviral effects of Glyh-101 were inferior to those of ACV. There were not enough data to evaluate the safety of Glyh-101 completely in vivo in this study. Therefore, more studies are needed to enhance and evaluate the efficacy and safety of CFTR inhibitors, as well as developing them into a potential novel treatment or a combination therapy with ACV against HSV infection.

The CFTR is a cAMP- sensitive Cl^−^ channel [9,32], and intracellular cAMP is generated from ATP by adenylyl cyclases (ACs) [29]. Our study demonstrated that HSV-1 and HSV-2 infection induced an up-regulation in the AC-cAMP signal that resulted in decreased intracellular Cl^−^ concentrations ([Cl^−^]_i_) in vitro. But, the specific mechanism by which HSV-1 and HSV-2 infection up-regulates ACs was not deeply explored in this study. HSV infection induced an increase in the intracellular calcium concentration ([Ca^2+^]_i_), which plays a critical role in facilitating viral infection [37,38]. Calcium could stimulate AC activity [39,40,41]. Therefore, we speculated that herpes simplex virus infection may up-regulate the expression and activity of ACs by increasing intracellular calcium. More studies are needed to prove this hypothesis. Additionally, we found that the concentration of AC continued to increase from 0.5 to 24 h post-infection. The concentration of intracellular cAMP only significantly increased from 0.5 to 4 h post-infection. However, after 4 h, the concentration of intracellular cAMP decreased but remained higher than before infection. We speculated that the reason for this might be that a cytosolic DNA sensor, cGAS, competed in order for the substrate ATP to produce more cyclic-GMP-AMP (cGAMP), which binds to and activates the adaptor protein STING, resulting in IRF3 activation and IFNβ induction at the late stage of infection [42].

The CFTR is a Cl^−^ channel and dynamically modulates the homeostasis of the intracellular Cl^−^ concentration ([Cl^−^]_i_) [9,32]. We observed that the [Cl^−^]_i_ concentration gradually decreased in HSV-1 and HSV-2 infected cells, and CFTR inhibitors increased the amount of [Cl^−^]_i_. Therefore, we pay more attention to Cl^−^-sensitive genes, such as GSK1, in terms of mechanism exploration. Firstly, higher extracellular concentrations of NaCl reportedly activated SGK1 [33,34]. Secondly, there is a mutual regulatory effect between the expression and activity of CFTR and SGK [43]. The loss of CFTR function itself in a CF lung epithelial cell line did not increase SGK1 expression [44]. The increase in salinity promoted the translocation of CFTRs from an intracellular pool to the plasma membrane, and that this effect may be mediated by SGK1 [45]. Thirdly, SGK1 had been reported to be involved in HSV infection. Stress can directly stimulate SGK1 levels as well as stimulate herpes simplex virus 1 (HSV-1) productive infection and reactivation from latency [46]. Therefore, the changes in SGK1 were primarily detected to explore the antiviral mechanism in this study. We believe that CFTR inhibitors suppressed the HSV infection through a variety of other target molecules. This necessitates further research to uncover the specific intracellular signaling underlying the antiviral effects of CFTR inhibition.

The regulation of CFTR expression and activity by different types of viruses was inconsistent. H5N1 HA attachment inhibited cAMP-dependent CFTR activity via JAK3-mediated adenylyl cyclase (AC) suppression, which reduces cAMP production [21]. HIV preserved the mRNA expression and function of the CFTR in airway epithelial cells [17]. BK polyomavirus (BKPyV) required CFTR activity to infect primary renal proximal tubular epithelial cells [13]. SARS-CoV-2 replication also required the expression and function of the CFTR. People with cystic fibrosis (CF), who have a mutation in the CFTR, were less susceptible to SARS-CoV-2 infection [14,47]. Our data indicated that HSV infection activated CFTR activity via increasing the expression of adenylyl cyclases (ACs), which induced cAMP production.

## 5. Conclusions

**Figure 7:**
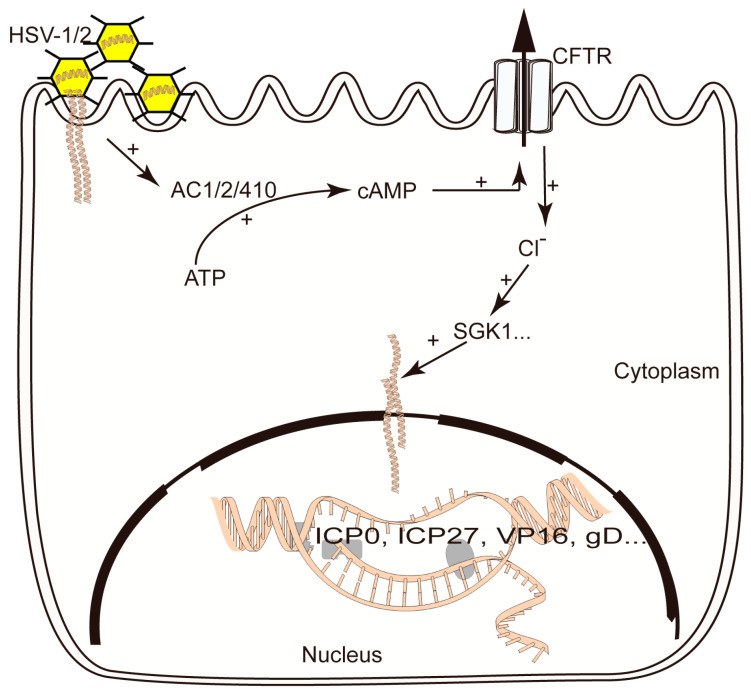
The proposed working model shows that CFTR inhibitors suppress HSV-1 and HSV-2 infection. CFTR inhibitors have an antiviral effect on HSV-1 and HSV-2 infection in vitro and in vivo. Herpes simplex virus infection could up-regulate the ACs-cAMP signal to activate CFTR activity, resulting in a decrease in [Cl^−^]_i_. The CFTR inhibitor suppressed the CFTR-mediated Cl^−^ transport activity to elevate [Cl^−^]_i_, resulting in decreased SGK1 activity in infected cells.

We proposed a model, shown in Figure 7. CFTR inhibitors have antiviral effects in vivo and in vitro. Herpes simplex virus infection could up-regulate the ACs-cAMP signal to activate CFTR activity resulting in a decrease in [Cl^−^]_i_. CFTR inhibitors suppressed the CFTR-mediated Cl^−^ transport activity to elevate [Cl^−^]_i_, resulting in decreased SGK1 activity in infected cells. CFTR inhibitors suppressed the expression and phosphorylation of serum and glucocorticoid-inducible protein kinase 1 (SGK1) in infected cells. We speculated that the antiviral effect of CFTR inhibitors partly relies on the change in SGK1. Our findings elucidated a previously unknown role of CFTR inhibitors in HSV-1 and HSV-2 infection.

## Figures and Tables

**Table 1:** The primers that were used in the quantitative PCR.

Factors	Forward	Reverse
GAPDH	CTCTGCTCCTCCTGTTCGAC	AGTTAAAAGCAGCCCTGGTGA
ICP0	GTGCATGAAGACCTGGATTCC	GGTCACGCCCACTATCAGGTA
ICP27	TGTCGGAGATCGACTACACG	GGTGCGTGTCCAGTATTTCA
gD	CCAAATACGCCTTAGCAGACC	CACAGTGATCGGGATGCTGG
VP16	AATGTGGTTTAGCTCCCGCA	CCAGTTGGCGTGTCTGTTTC
CFTR	TGCCCTTCGGCGATGTTT	GCGATAGAGCGTTCCTCCTTG
SGK1	GGTGGCAATTCTCATCGCTT	GGCCAAGGTTGATTTGCTGA

## Data Availability

Data are contained within the article.
